# Utility and optimal cut-off point of the Somatic Symptom Scale-8 for central sensitization syndrome among outpatients with somatic symptoms and related disorders

**DOI:** 10.1186/s13030-022-00253-2

**Published:** 2022-11-22

**Authors:** Kazuaki Hashimoto, Takeaki Takeuchi, Miki Hiiragi, Akiko Koyama, Yuzo Nakamura, Masahiro Hashizume

**Affiliations:** Department of Psychosomatic Medicine, Toho University School of Medicine, 6-11-1 Omori-Nishi, Ota-Ku 143-8541 Tokyo, Japan

**Keywords:** Central sensitization syndrome, Somatic symptom disorder, Somatic Symptom Scale-8

## Abstract

**Background:**

Central sensitization syndrome (CSS) involves severe functional symptoms due to central sensitization. for patients with severe somatic symptoms and related disorders (SSRDs), central sensitization may be responsible for their functional symptoms. We hypothesized that screening for CSS in patients with SSRDs would identify those with severe disease. The Somatic Symptom Scale-8 (SSS-8) is a simple tool to assess medical conditions related to SSRDs, but the cut-off point to identify severe cases of comorbid CSS is unknown. This study aimed to determine the optimal cut-off point of SSS-8 for screening the CSS of patients with severe SSRDs.

**Methods:**

In total, 143 patients with SSRDs attending outpatient clinics of a university hospital in Japan were included in the study. The participants were evaluated using the SSS-8 for somatic symptoms, Hospital Anxiety and Depression Scale (HADS) for anxiety and depressive symptoms, Pain Catastrophizing Scale (PCS) for catastrophic thoughts, and Central Sensitization Inventory (CSI-A, B) for CSS. Receiver operating characteristic (ROC) curve analysis was performed using the propensity score. The area under the curve (AUC) was calculated using a propensity score considering PCS, age, sex, HADS, and CSI-B as confounders of SSS-8 and CSS to evaluate differences in diagnostic accuracy between patients with and without SSS-8. The sensitivity and specificity of the ROC analysis were then used to determine the cut-off point for discriminating severe cases of SSS-8.

**Results:**

Of the 143 participants, 126 responded (51 CSS group and 75 non-CSS group), with a valid response rate of 88.1 percent. In the ROC analysis, the propensity score including SSS-8 was statistically more accurate. The optimal cut-off point was 13, with an AUC of 0.88, sensitivity of 84.3 percent, and specificity of 77.3 percent.

**Conclusions:**

The SSS-8 is a useful tool for discriminating severe cases of SSRDs comorbid with CSS.

## Background

The somatic symptoms observed in patients with somatic symptom and related disorders (SSRDs) [1] are not based on fatal organic abnormalities [2] and are considered to reflect a complex syndrome of biological, psychological, and social problems [3]. Patients with SSRDs commonly present with multiple somatic symptoms, and the Patient Health Questionnaire-15 (PHQ-15) is used to assess these symptoms [4]. Additionally, the Somatic Symptom Scale-8 (SSS-8) was developed to more easily assess the disease status of SSRDs [5]. The SSS-8 was developed as a short form of the PHQ-15, and is used in clinical practice for follow-up of medical conditions in primary care [6] and psychosomatic outpatient clinics [7].

In patients with SSRDs, pharmacotherapy and psychotherapy have been attempted to treat the somatic symptoms and psychological problems of the patients [8]. However, in such patients, functional somatic symptoms typically persist for more than 6 months [1] and are particularly difficult to treat in severe cases [9, 10]. A previous study has reported that functional somatic symptoms are most severe when affected by central sensitization in particular [11, 12]. Central sensitization is defined as a neurophysiological condition in which hyperexcitability of the central nervous system induces hyperalgesia [13] and affects psychological factors such as catastrophic thoughts [14]. Conditions that are strongly influenced by central sensitization are comprehensively treated as central sensitization syndrome (CSS) [13].

Hence, screening for CSS in patients with SSRDs would be useful for identifying those with severe SSRDs. The severity of SSRDs are classified into five levels when using the SSS-8 scores [5]. However, in busy clinical practice, the SSS-8 would serve as a more convenient screening tool by setting a cut-off point for the SSS-8 to discriminate severe cases. The aim of this study was to determine the optimal cut-off point of SSS-8 for the screening of CSS among patients with severe SSRDs.

## Methods

### Participants

The study was cross-sectional. Participants were recruited from among the patients who visited the Department of Psychosomatic Medicine at Toho University Medical Center Omori Hospital between February and March 2021. The inclusion criteria were as follows: 1) age 20–79 years; 2) accurate understanding of the purpose and process of the study and signing an informed consent form; 3) meeting the diagnostic criteria for SSRDs [1]. Exclusion criteria included diagnosis of 1) schizophrenia spectrum disorder and other psychotic disorders; 2) dementia (such as Alzheimer's dementia, vascular dementia, Parkinson's disease dementia, and Lewy body dementia); 3) neurodevelopmental disorders (such as autism spectrum disorder, attention deficit/hyperactivity disorder, communication disabilities); 4) dissociative disorders; and 5) patients who for any reason could not be accurately assessed.

The diagnoses were made by multiple physicians using the Diagnostic and Statistical Manual of Mental Disorders, 5th ed. (DSM-5) [1]. Data regarding age, sex, education, and duration of treatment were collected as background factors from all participants.

### Questionnaires

The SSS-8 [5] was used to assess somatic symptoms; the Japanese version of the SSS-8 [15] has been validated linguistically and psychologically and has internal consistency [16].

The Central Sensitization Inventory (CSI) [17] was used to assess central sensitization. The CSI consists of two parts. Part A assesses subjective symptoms common to CSS and Part B asks whether the subject has had CSS in the past. CSI is a questionnaire with high reliability and internal consistency, and the reliability and validity of the Japanese version of CSI have already been verified in a previous study [18]. The CSI correlates with quantitative sensory tests used for inferring CSS [19, 20], and a cut-off point of 40 or higher on the CSI-A has been reported to be useful for discriminating CSS in outpatient clinics [21]. In this study, patients with a CSI-A of 40 points or higher were included in the CSS group.

We assessed the participants' state of anxiety, depression, and catastrophic thinking, which are psychological states that have been reported to be related to central sensitization in previous studies [22–24].

The Hospital Anxiety and Depression Scale (HADS) [25] is a questionnaire consisting of seven items each on anxiety and depression. Both the anxiety and depression scales are scored from 0 to 21 points and are used as clinical indicators of psychiatric symptoms in general practice [26]. The HADS has also been reported to be associated with quality of life [27], and the Japanese version of the HADS has been validated for reliability and validity [28]. The Pain Catastrophizing Scale (PCS) [29] is a 13-item questionnaire with three subscales (rumination, helplessness, and magnification) that assesses catastrophic thinking and has shown high reliability and validity. The reliability and validity of the Japanese version of the PCS were also confirmed [30].

### Data analysis

For differences in background factors and endpoints between the CSS and non-CSS groups, nominal variables were subjected to chi-square or Fisher’s test, continuous variables to t-test, and categorical variables and non-normally distributed continuous variables to Mann–Whitney U test.

To evaluate the utility of the SSS-8 in discriminating between the CSS and non-CSS groups, two propensity scores were calculated by logistic regression analysis. One was the propensity score with CSS as the dependent variable, SSS-8 as the independent variable, and PCS, HADS, age, sex, and CSI-B as confounders of CSS, and the other was the propensity score with CSS as the dependent variable and PCS, HADS, age, sex, and CSI-B as independent variables. Receiver operating characteristic (ROC) curve analyses were performed on the propensity scores [31] to statistically compare the area under the curve (AUC) with and without SSS-8 as an independent variable. The optimal cut-off point of SSS-8 was determined by the Youden Index to distinguish the group with severe CSS, and the accuracy of the test was evaluated by its sensitivity and specificity.

All analyses in this study were performed using EZR Version 1.32 [32]. Two-tailed *P*-values less than 0.05 were considered statistically significant.

## Results

Of the 143 participants who met the criteria for this study, 17 were excluded because of missing data or inappropriate responses, leaving the data of 126 available for analysis. The valid response rate was 88.1 percent.

Fifty-one participants were included in the CSS group, defined by a CSI-A of 40 points or higher, and 75 participants were included in the non-CSS group. Table 1 shows a comparison of the data of the two groups: there were more females in the CSS group than in the non-CSS group, the mean age was lower, and more of the patients had a history of CSS. Additionally, the CSS group had significantly higher scores on the HADS anxiety and depression scales, CSI-A, PCS, and SSS-8 than the non-CSS group.

**Table 1:** Patient Characteristics (*n* = 126)

	Non-CSS (*n* = 75)	CSS (*n* = 51)	*P* value
Sex			< 0.01
Male	34(45.3%)	9(17.6%)	
Female	41(54.7%)	42(82.4%)	
Age(years)	60.0 [22.0–81.0]	49.0 [26.0–83.0]	< 0.01
Education(years)	14.0 [ 9.0 -20.0]	14.0 [ 9.0 -16.0]	0.86
Treatment duration(months)	36.0 [3.0–204.0]	66.0 [3.0–204.0]	0.53
Questionnaire			
CSI-A	23.0 [1.0–38.0]	49.0 [40.0–90.0]	< 0.001
CSI-B	1.0 [ 0.0 -4.0]	2.0 [ 0.0 -6.0]	< 0.001
SSS-8	9.0 [1.0–24.0]	17.0 [6.0–32.0]	< 0.001
HADS Anxiety	5.0 [0.0–15.0]	10.0 [3.0–19.0]	< 0.001
HADS Depression	5.0 [0.0–15.0]	9.0 [1.0–17.0]	< 0.001
PCS	19.0 [0.0–49.0]	35.0 [0.0–52.0]	< 0.001

*CSI* Central Sensitization Inventory, *SSS-8* The Somatic Symptom Scale-8, *HAD* Hospital Anxiety and Depression Scale, *PCS* Pain Catastrophizing Scale

**Fig. 1:**
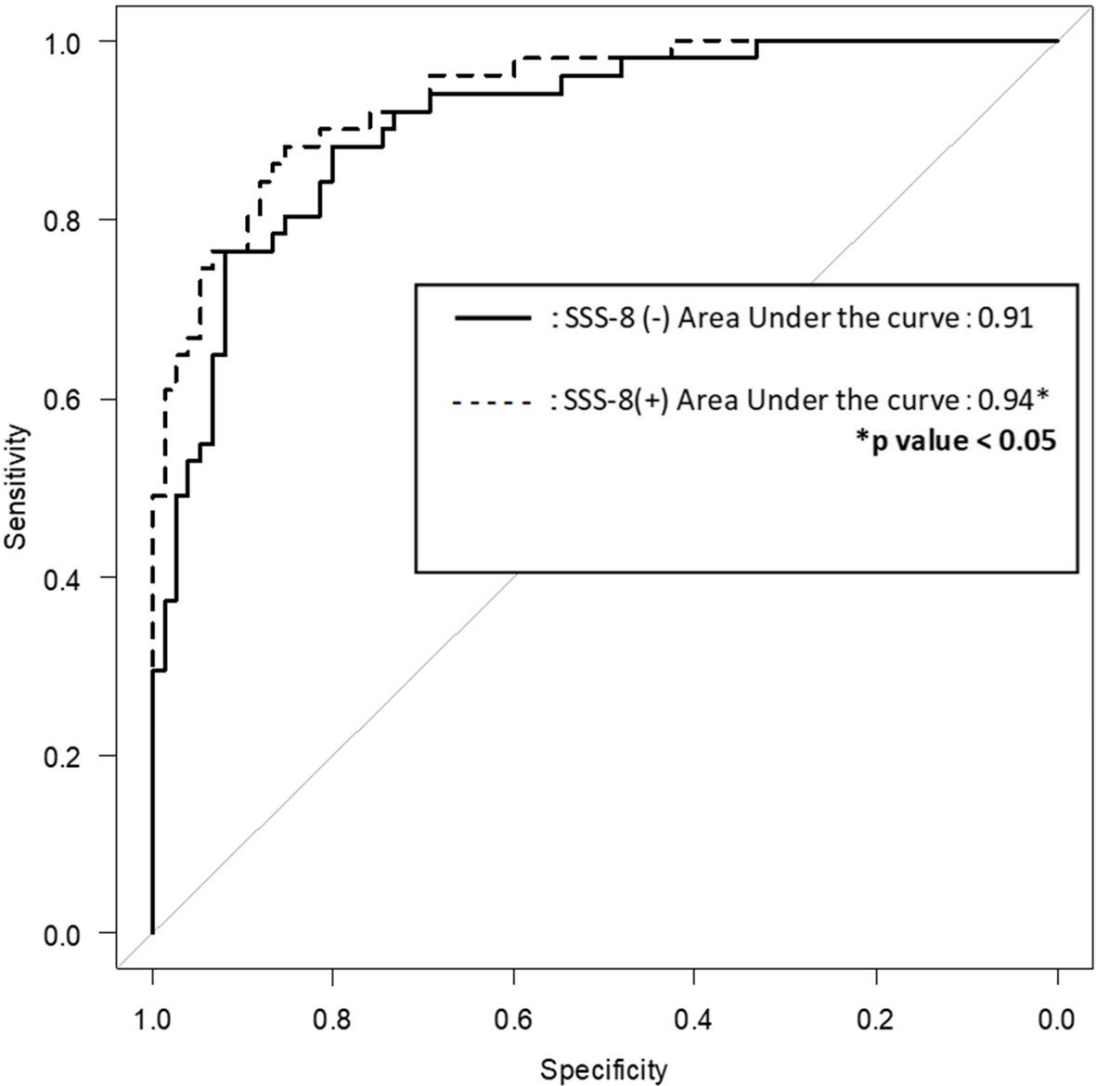
Comparison of discrimination accuracy for central sensitization syndrome between the Somatic Symptom Scale-8 (SSS-8) (+ ; dashed line) and SSS-8 (-; solid line) among the somatic symptom and related disorders patients (*n* = 126)

Figure 1 shows a comparison of the AUCs for a propensity score including SSS-8 and a propensity score not including SSS-8 for discrimination between the CSS and non-CSS groups. Both AUCs were above 0.7, but the AUC of the propensity score including SSS-8 was significantly larger than that of the propensity score not including SSS-8 (*p* < 0.05).

**Fig. 2:**
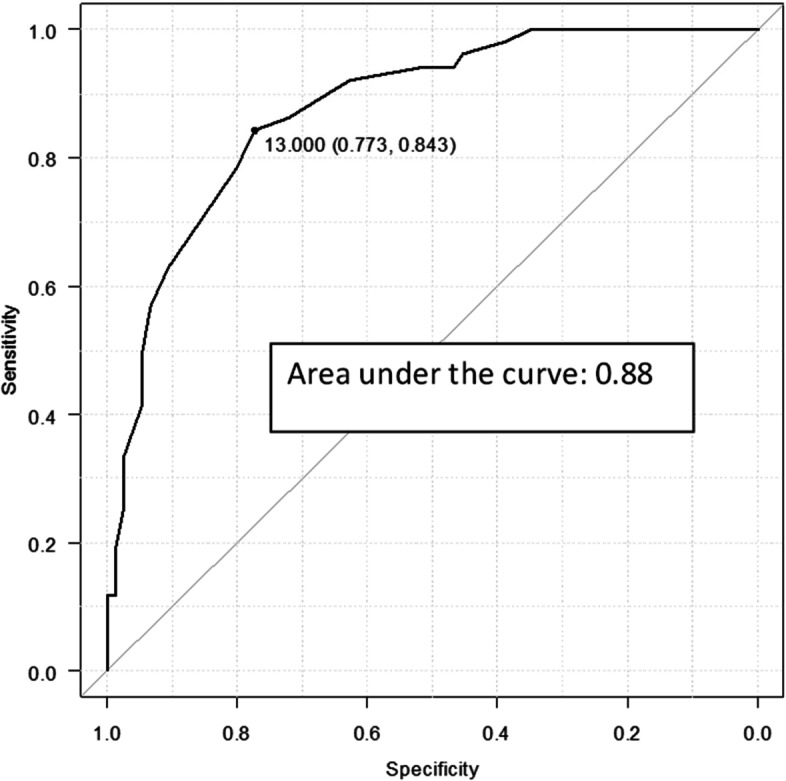
Receiver operating characteristic curve to determine the cut-off points on the Somatic Symptom Scale-8 for discriminating central sensitization syndrome among the somatic symptoms and related disorders patients (*n* = 126)

Figure 2 shows the ROC curve and cut-off point for the screening patients with severe SSS-8, and Table 2 shows a summary of various cut-off point scores. The optimal SSS-8 cut-off point using the Youden index was 13 points, sensitivity was 84.3 percent, specificity was 77.3 percent, and the AUC was 0.88.

**Table 2:** Summary of the cut points scores on the Somatic Symptom Scale-8 to discriminate the central sensitization syndrome among the somatic symptoms and related disorders patients (*n* = 126)

Cut point score	specificity [95%CI]	sensitivity [95%CI]	positive predictive value [95%CI]	negative predictive value [95%CI]
10	0.52 [0.40–0.64]	0.94 [0.84–0.99]	0.57 [0.46–0.68]	0.93 [0.81–0.99]
11	0.63 [0.51–0.74]	0.92 [0.81–0.98]	0.63 [0.51–0.74]	0.92 [0.81–0.98]
12	0.72 [0.60–0.82]	0.86 [0.74–0.94]	0.68 [0.55–0.79]	0.89 [0.78–0.95]
13	0.77 [0.66–0.86]	0.84 [0.71–0.93]	0.72 [0.59–0.83]	0.88 [0.78–0.95]
14	0.80 [0.69–0.88]	0.78 [0.65–0.89]	0.73 [0.59–0.84]	0.85 [0.74–0.92]
15	0.85 [0.75–0.92]	0.71 [0.56–0.83]	0.77 [0.62–0.88]	0.81 [0.71–0.89]
16	0.91 [0.82–0.96]	0.63 [0.48–0.76]	0.82 [0.67–0.93]	0.78 [0.68–0.86]
17	0.93 [0.85–0.98]	0.57 [0.42–0.71]	0.85 [0.69–0.95]	0.76 [0.66–0.84]

*CI* confidence interval

## Discussion

In this study, we examined the utility of the SSS-8 with an optimally chosen cut-off point for discriminating patients with severe disease comorbid with CSS in patients with SSRDs. The SSS-8 was useful for discriminating severity even when confounding factors were considered, and the accuracy of the test was high when the cut-off point was set at 13 or higher.

Generally, sex differences exist in pain sensitivity, and it has been reported that women have lower pain thresholds than men as a biological characteristic [33]. Furthermore, many conditions that fall under CSS are known to be more frequent in women [21, 34–36]. In this sample, there were more women in the CSS group, which is consistent with the characterizations of previous reports. Additionally, the prevalence of CSS conditions tends to decrease with age, for example migraine [37], and the prevalence of irritable bowel syndrome is also low in adults > 50 years of age according to a worldwide meta-analysis [38]. In the present study, the CSS group was younger than the non-CSS group, which is consistent with the trends found in previous studies [37, 38].

The SSS-8 can be used to assist in the diagnosis of somatic symptomatology according to DSM-5 [1] and is useful in assessing clinical severity [39]. A total score of 12 points or higher on the German version of the SSS-8 was considered to be a high somatic symptoms burden on the patient, and scores were divided into five levels of 4 points each [5], yielding three levels of mild to moderate symptoms and two levels of severe cases. In our study, we determined a cut-off value of 13 points on the SSS-8 for discriminating severe cases, which is similar to that in a previous study [5]. The value of the AUC of the propensity score without inclusion of SSS-8 as a variable was 0.91. Therefore, even without using the SSS-8, it may be possible to discriminate severe conditions of SSRDs with high accuracy by just integrating information on background factors such as age, sex, and levels of anxiety, depression, and catastrophic thoughts. However, in our results, the SSS-8 score was found to further improve the accuracy of discriminating severe cases and thus can be a useful tool for screening. Most patients with SSRDs have a high level of functional impairment [40], but no abnormalities are found in biological tests [2]. Therefore, patients often feel anxious about their medically unexplained symptoms and frequently seek explanations from their health care providers [41]. The development of a cut-off point for the SSS-8 will help link the presence or absence of CSS to the intensity of unexplained somatic symptoms, which will provide anxiety relief to patients in the severe group, making it a useful clinical indicator.

### Strengths and limitations

This study is clinically meaningful in that it proposes an index for rapid identification of severe symptoms and related disorders. However, there are some limitations to the interpretation. First, we defined the presence of central sensitization syndrome using a questionnaire with CSI. According to previous studies [21, 42], the assessment of central sensitization with the CSI is very precise, but in the present study we did not directly extract physiological changes, and it is unclear how the history of CSS was diagnosed. Second, although the sample size was large enough to reach statistically significant results [43], the sample size was limited by the fact that it was a single site study. Third, the participants may have been better educated than the general SSRDs group [1], and effects of medication and treatment history were not considered. Hence, although our results approximate those of the general population [5], full generalizability cannot be assumed.

## Conclusions

In conclusion, this study reported that the SSS-8 was a useful tool for the discrimination of severe cases of SSRDs. We found the optimal cut-off point for this discrimination an SSS-8 score of 13 points.

## Data Availability

We are not able to share our data because sharing data is not permitted by our hospital ethics committees.
